# Hypertrophic Lichen Planus and Hypertrophic Skin Lesions Associated with Histological Lichenoid Infiltrate: A Case Report and Literature Review

**DOI:** 10.3390/dermatopathology12010008

**Published:** 2025-02-25

**Authors:** Biagio Scotti, Cosimo Misciali, Federico Bardazzi, Bianca Maria Piraccini, Michelangelo La Placa

**Affiliations:** 1 Dermatology Unit, IRCCS Azienda-Ospedaliero Universitaria di Bologna, 33-40126 Bologna, Italy; biagio.scotti2@unibo.it (B.S.); cosimo.misciali@aosp.bo.it (C.M.); federico.bardazzi@aosp.bo.it (F.B.); biancamaria.piraccini@unibo.it (B.M.P.); 2 Department of Medical and Surgical Sciences, Alma Mater Studiorum University of Bologna, 33-40126 Bologna, Italy

**Keywords:** hypertrophic lichen planus, HLP, hypertrophic lichenoid dermatitis, hypertrophic lichen sclerosus, lichen simplex chronicus, squamous cell carcinoma, pseudoepitheliomatous hyperplasia, viral wart, HPV, lupus erythematosus/lichen planus overlap syndrome, LE/LP overlap, histology, histopathology

## Abstract

Hypertrophic lichen planus (HLP) is a chronic inflammatory skin condition defined by verrucous, pruritic, papules and plaques usually affecting the lower limbs. The diagnosis of HLP is primarily clinical. However, due to its feasible generalized presentation and similarities with other hypertrophic cutaneous disorders, histological evaluation is often necessary. Many dermatological conditions that present with a hypertrophic clinical appearance can arise from a histological lichenoid infiltrate (HCLI). Hence, we provide an overview of the clinical, histopathological, and prognostic features of selected HCLI, including HLP, hypertrophic lichenoid dermatitis, hypertrophic lichen sclerosus (HLS), lichen simplex chronicus (LSC), squamous cell carcinoma (SCC), keratoacanthoma (KA), pseudoepitheliomatous hyperplasia (PEH), viral warts, and lupus erythematosus/lichen planus (LE/LP) overlap. Choosing the appropriate procedure and the anatomical site for an incisional biopsy requires thoughtful consideration to ensure sufficient depth and improve diagnostic accuracy by identifying the histological features specific to each hypertrophic condition.

## 1. Introduction

Lichen planus (LP) is an inflammatory disorder characterized by an idiopathic T-cell-mediated process that affects the skin, mucous membranes, and other ectodermal-derived tissues [1,2]. Cutaneous LP occurs in less than 1% of the general population, without a strong or well-established gender predilection [3]. It typically presents as a papulosquamous eruption with flat-topped, violaceous lesions often described using the “six P’s” (papules, plaques, purple, pruritic, polygonal, and planar) and characterized by the classic Wickham striae, fine white or grey lines mostly described on mucosal sites [3]. Variants of LP include hypertrophic, atrophic, annular, vesicular-bullous, actinic, pigmentosus, follicular, inverse, porokeratotic, and ulcerative [4,5]. The distribution of LP is primarily reported on the extremities, with the hypertrophic form most commonly affecting the lower limbs and interphalangeal joints, while generalized presentations are rare [6]. Together with the clinical aspects, the histology allows a reliable diagnosis and distinction between the different subtypes of lichen. Studies in the literature focusing on the histological features of hypertrophic lichen planus (HLP) are limited and often fragmented. Nevertheless, distinct hypertrophic dermatoses may be associated with a variable lichenoid, band-like infiltrate. Starting from the presented report, our review aims to highlight the clinical, histological, and prognostic distinctions among the primary dermatological conditions characterized by both a hypertrophic clinical appearance and a lichenoid infiltrate (HCLI) on histopathology. This insight will help clinicians make accurate differential diagnoses and establish prognoses for each hypertrophic condition.

## 2. Materials and Methods

A narrative review was conducted starting from Medline (PubMed) library and following the principles of evidence-based medicine. The keyword “hypertrophic lichen planus” returned 242 results, while the combination of (hypertrophic lesions AND lichen planus) yielded 83 results. An additional search was performed for “hypertrophic lesions” along with specific dermatological conditions that may be associated with a dermal band-like inflammatory infiltrate: squamous cell carcinoma (SCC), keratoacanthoma (KA), lichen simplex chronicus (LSC), pseudoepitheliomatous hyperplasia (PEH), human papilloma virus (HPV), and lupus erythematosus (LE). This review aim to explore the clinical, histopathological, and prognostic differences among the hypertrophic skin lesions that may arise from a histological lichenoid infiltrate (HCLI).

## 3. Results

### 3.1. Case Report

A 60-year-old man from Sri Lanka with a history of chronic kidney disease and non-alcoholic steatohepatitis (NASH) presented to our Dermatology department with a 1-year record of widespread, pruritic, warty lesions covering most of his body. The skin manifestations initially appeared on his right ankle and progressively spread to his legs, arms, and trunk.

**Figure 1:**
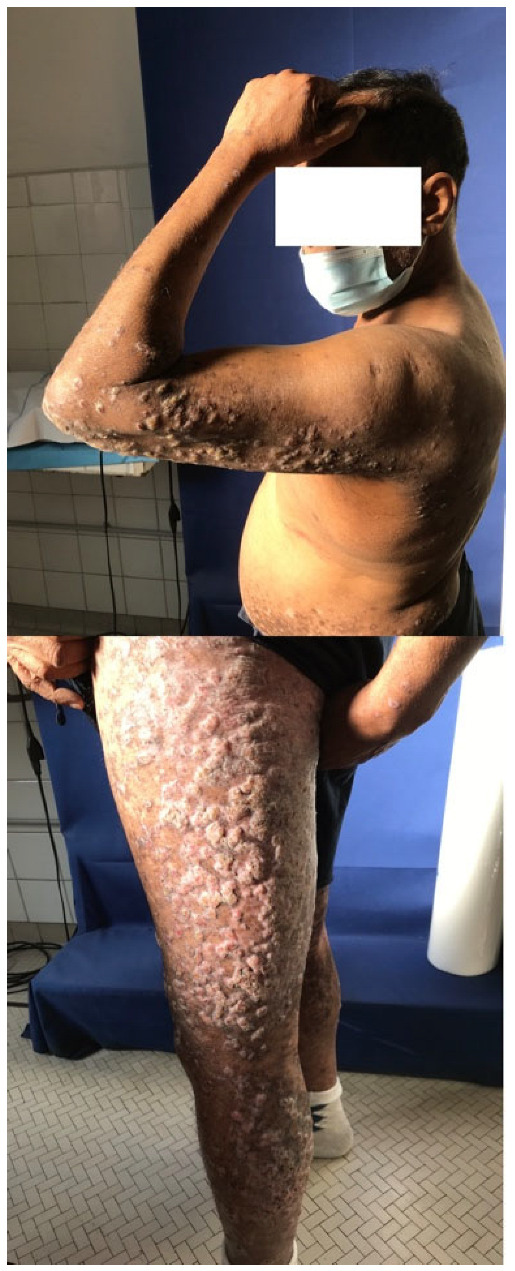
Clinical pre-treatment assessment. Diffuse hypertrophic, erythematous-pinkish papules and plaques, mainly located on the upper and lower extremities.

**Figure 2:**
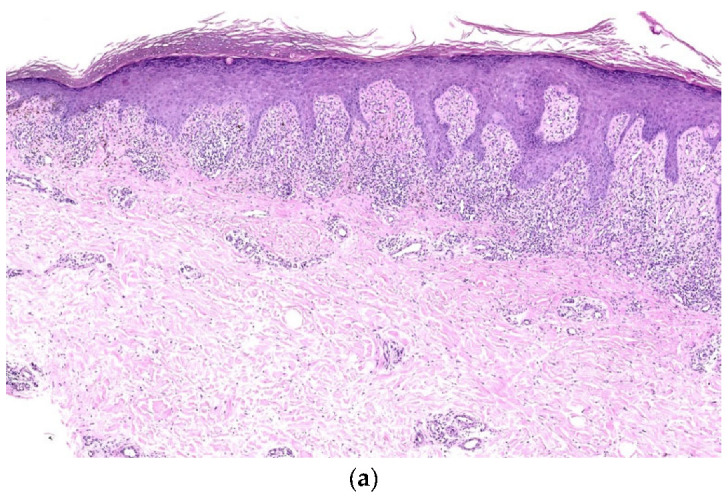

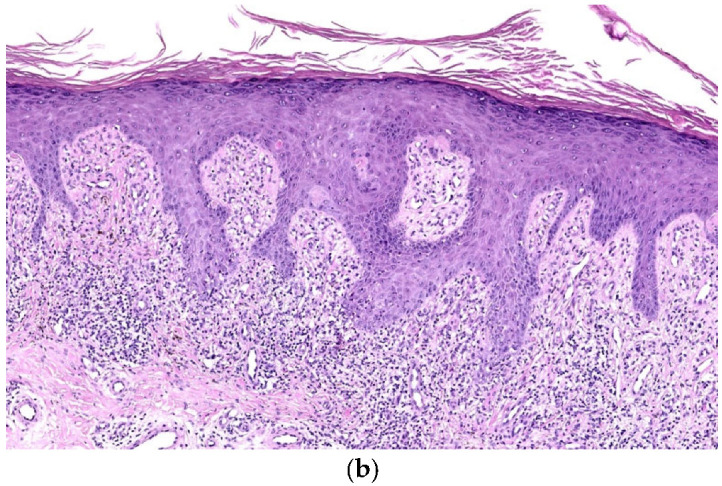
(**a**) Lichen simplex chronicus (LSC) superimposed on lichen planus (LP), histopathology (Hematoxylin-eosin stain 3×) (H&E stain 3×). (**b**) LSC superimposed on LP, histopathology (detail). Compact, basket-woven orthokeratosis, foci of hypergranulosis, epidermal hyperplasia, edema, dilated blood vessels, and dense and band-like infiltrate of lymphocytes in the thickened papillary dermis obscuring the DEJ (Hematoxylin-eosin stain 8×) (H&E stain 8×).

On clinical examination, multiple hypertrophic papules, partially coalescing into plaques, were observed on the upper and lower limbs, buttocks, and lower abdomen. The plaques were well-defined, scaly, purple, and exhibited a verrucous appearance. Several lesions were excoriated and covered with crusts (Figure 1). No mucosal lesions were detected. A punch biopsy was performed on the thigh, revealing compact, basket-weave orthokeratosis, areas of hypergranulosis, necrotic keratinocytes, and irregular epidermal hyperplasia. Additionally, oedema, dilated blood vessels, and a dense, band-like infiltration of lymphocytes were detected in the thickened and regular/irregular papillary dermis, obscuring the dermoepidemal junction (DEJ) (Figure 2a,b).

Based on the clinical and histological findings, a final diagnosis of lichen planus (LP) with superimposed lichen simplex chronicus (LSC) was made. Despite the patient’s comorbidities, we initiated treatment with triamcinolone acetonide 40 mg/mL, administered intramuscularly every 20 days for a total of three injections. A topical regimen of mometasone furoate and a keratolytic gel, applied twice daily, was also prescribed. Given the severity of pruritus, amitriptyline hydrochloride was added to the treatment plan. After 5 weeks, the patient’s lesions and symptoms showed significant improvement.

### 3.2. Hypertrophic Clinical Skin Lesions with Histological Lichenoid Infiltrate (HCLI)

The lichenoid infiltrate is characterized by a dense, continuous, band-like lymphohistiocytic infiltrate at the dermoepidermal junction (DEJ). Several conditions were considered for differential diagnosis among the HCLI, including hypertrophic lichen planus (HLP), hypertrophic lichen sclerosus (HLS), hypertrophic lichenoid dermatitis, squamous cell carcinoma (SCC), keratoacanthoma (KA), lichen simplex chronicus (LSC), pseudoepitheliomatous hyperplasia (PEH), viral warts, and lupus erythematosus/lichen planus (LE/LP) overlap syndrome. The clinical, histopathological, and prognostic data for HCLI are summarized in Table 1.

#### 3.2.1. Hypertrophic Lichen Planus (HLP)

Hypertrophic lichen planus (HLP), also known as lichen planus verrucosus or lichen planus hypertrophicus, is a distinct variant of LP [7]. It typically presents with *pinkish* to *purple-red* or *bluish hyperkeratotic* papules, plaques, and nodules variably distributed across the body, but commonly seen on the lower extremities, where it is closely associated with chronic venous insufficiency [8]. HLP, with its characteristic verrucous appearance, may resemble many other dermatological conditions [9], although differing significantly from the other subtypes of LP: (a) Classical and atrophic LP tends to be more generalized, with thinner plaques or papules and less prominent hyperkeratosis. (b) Erosive/ulcerative and vesicular-bullous LP are characterized by ulcers and blisters, mainly affecting mucosal surfaces like the oral mucosa and genitalia, often causing discomfort and potentially scarring. (c) Annular LP presents as ring-shaped lesions with central clearing and raised borders, commonly affecting the penis and axilla, typically appearing less pruritic than the hypertrophic form. (d) Actinic LP presents as erythematous or hyperpigmented patches, usually affecting the sun-exposed areas of darker phototype individuals. (e) LP pigmentosus is characterized by greyish-brown or darkly pigmented patches, primarily involving the face, neck, and upper body. (f) Follicular LP (lichen planopilaris) regards the hair follicles, causing scarring alopecia with perifollicular erythema, scaling, and papules. (g) Porokeratotic LP lacks the typical clinical lichenoid features, instead presenting as hyperpigmented papules or plaques with an annular configuration, accompanied by focal parakeratosis and *cornoid* lamella observed on histology or reflectance confocal microscopy. (h) Inverse LP occurs in skin folds, such as the axillae, groin, and submammary region, where lesions appear in moist, occluded areas, in contrast to the more typical acral sites of LP [3,4,5,6].

Although less commonly described, HLP can affect the genitalia and perineal area, resulting in hyperkeratotic white plaques, particularly in women during their 5th or 6th decade of life and rarely in young adults [10,11,12,13]. Among the variants of LP that can involve these sensitive anatomical areas, HLP may present with severe clinical manifestations, often requiring treatments such as phototherapy (narrow-band ultraviolet B radiation or psoralen plus ultraviolet A photochemotherapy) or steroid-sparing systemic agents as initial therapy [3,14,15].

Though extremely rare, when HLP presents with a whitish verrucous appearance, it must be differentiated from hypertrophic lichen sclerosus (HLS), a rare subtype of LS with distinct features, including both atrophic areas and verrucous hyperplasia on the surface. This variant is typically seen in the extragenital area and presents with the classic histological features of LS, such as hyperkeratosis, thinned and effaced epidermis, a broad band of hyalinization in the upper dermis, and a lichenoid infiltrate beneath [16]. However, early-stage HLS may be characterized by the *absence of sclerosis* and a lymphocytic infiltrate located immediately beneath the epidermis rather than being displaced downward, making differential diagnosis challenging. Despite signs of hyalinization, according to Weyers et al., the presence of lymphocytes scattered throughout the epidermis, along with psoriasiform hyperplasia, serves as a key distinguishing feature from LP [17].

In terms of prognosis, HLP generally follows a chronic course [18,19,20], and occasional remissions have been reported in association with hepatitis C virus (HCV) infection and autoimmune disorders [4]. Although most studies agree on the clinical presentation of this dermatological condition, the histopathology of HLP is often inconclusive for a defined diagnosis [21]. Classic LP is typically characterized by a dense lichenoid infiltrate with multiple apoptotic cells or colloid-hyaline (Civatte) bodies at the dermoepidemal junction. Epidermal changes usually include hyperkeratosis, hypergranulosis, and elongation of the rete ridges [8,9]. While parakeratosis and eosinophils are generally absent—helping to distinguish LP from lichenoid drug reactions—in about 20% of cases, a separation of the epidermis into small clefts (Max Joseph cleft formation) can be seen, as well as eosinophils in HLP [22]. 

**Figure 3:**
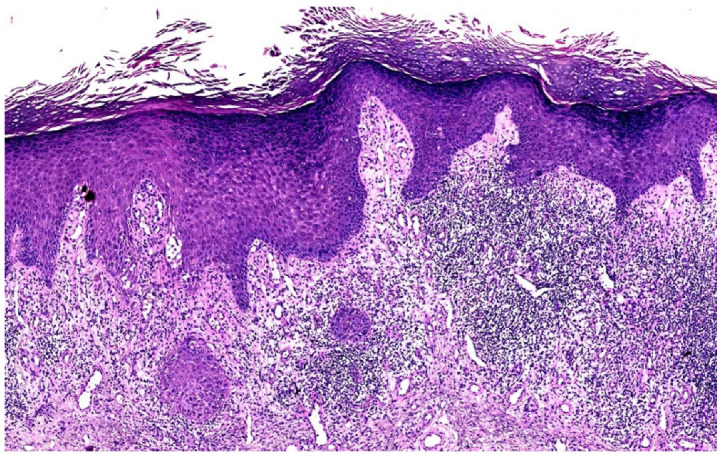
HLP histopathology. Hyperkeratosis, wedge-shaped hypergranulosis, infundibular hyperplasia of epidermis, thickened collagen fibers in the papillary dermis, and focal lymphocyte lichenoid infiltrate at the base of the infundibula (H&E stain 8×).

The histological variability of HLP arises from both the nature of the dermatitis, which sometimes lacks hypergranulosis and basal cell vacuolar degeneration [23], and the limitations of published studies where some histological aspects, such as the type of hypergranulosis (whether wedge-shaped or not) and the extent of epidermal thickening, are not always specified. Nevertheless, key histological features should be considered for differential diagnosis, including *wedge-shaped* hypergranulosis, endophytic well-differentiated squamous epithelium extending as deep as the superficial dermis, and irregular morphology of the papillary ridges [1,2,11] (Figure 3). The extent of the lichenoid infiltrate may vary, from focal involvement at the rete ridges to multifocal presence throughout the papillary dermis. Additionally, when squamous atypia is detected, it is typically graded as mild [24].

In cases of hypertrophic lichenoid dermatitis (HLD), the clinical and histopathological presentation may resemble that of HLP, with information about drug use (e.g., Pembrolizumab) necessary to make a distinction. To date, cases of HLD have been reported as immune-related adverse events (irAEs) in patients receiving immune checkpoint inhibitors [25,26,27]. These reactions often show nonclassic lichenoid features, with HLD lesions demonstrating more prominent spongiosis and eosinophilic infiltration, which are not typical of LP or HLP, and may present with a more generalized distribution, including the extremities and trunk.

#### 3.2.2. Squamous Cell Carcinoma (SCC)

Cutaneous squamous cell carcinoma (SCC) accounts for approximately 20% of all keratinocyte carcinomas, which are the most common form of cancer worldwide [27]. The neoplastic transformation of LP’ lesions is extremely rare, with the incidence of SCCs complicating LP estimated at about 0.4%, primarily affecting the hypertrophic subtype. This transformation typically occurs in longstanding, nonhealing, itchy lesions, usually located on the lower limbs or genitalia [28,29], and is more frequently observed in men than in women [30].

Although prior data suggest that SCC arising from hypertrophic lichen planus (HLP) generally carries a favorable prognosis with various treatment options, including conservative approaches [24,31], one case of metastatic SCC originating from malignant degeneration of HLP has been reported [32]. Additionally, some authors propose that the appearance of depigmented areas within LP lesions may serve as a clinical indicator of neoplastic transformation [20]. Nonetheless, this suggestion is not consistently supported [28], and LP is not currently considered a premalignant skin or mucosal condition [20].

Regarding mucosal involvement, oral LP (oLP) affects 15–30% of patients diagnosed with LP and is associated with cutaneous manifestations in 20–34% [33]. oLP carries a higher risk of malignant degeneration (0.4–15%) compared to its cutaneous counterpart. After oral involvement, the genitalia are the second most common mucosal site affected in LP [33,34]. In the context of oLP, squamous cell carcinoma (SCC) presents similarly to conventional SCC, typically as a *whitish tumor* with ulceration and elevated, indurated borders [35,36,37]. Both exophytic and endophytic growth patterns may be observed, leading to subsequent ulceration [36]. On the other hand, SCC in cLP typically appears as *erythematous-violaceous*, *hyperkeratotic*, and pruritic plaques with a localized distribution [30,38]. Histologically, SCC that develops in the context of cLP is indistinguishable from well-differentiated SCC, likely a variant of it, with a different clinical behavior. Keratoacanthoma (KA), presenting as a dome-shaped papule with a central keratotic plug, may also be associated with LP and, in extremely rare cases, can arise within LP or HLP [39]. The unique clinical course of KA, marked by rapid growth over weeks to months followed by spontaneous regression, along with the absence of asymmetry, irregularities in the central keratin plug, overhanging epithelial margins, or variations in the squamous cell organization, helps distinguish it in differential diagnosis [40]. However, perineural invasion and intravascular spread have also been reported in KA, meaning these features alone are not necessarily conclusive for malignancy. 

Biological mechanisms explaining the possible malignant transformation of LP to SCC have been proposed. Similar to other neoplasms that develop in the context of chronic inflammation, such as colorectal carcinoma in patients with chronic inflammatory bowel disease (IBD) [40], the inflammatory infiltrate in LP may induce oxidative stress and stimulate the release of inflammatory cytokines [41,42]. This process can modulate cell proliferation, differentiation, contributing to malignant transformation.

**Figure 4:**
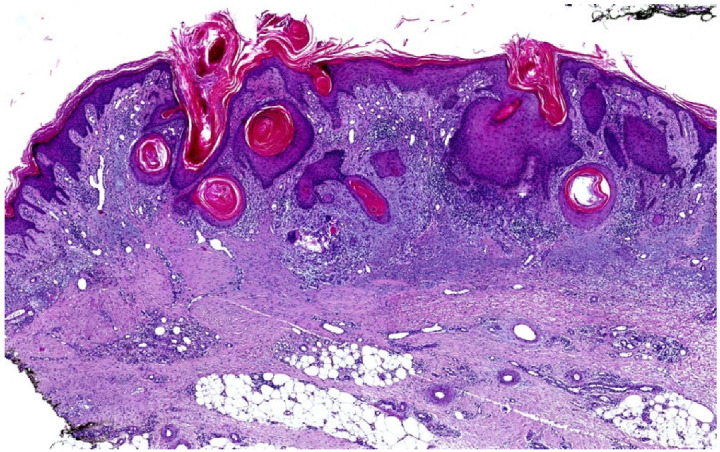
SCC arising from HLP histopathology. Hyperkeratosis, focal hypergranulosis, necrotic keratinocytes in the epidermis, vacuolar degeneration at the DEJ, solar elastosis, thickening of collagen fibers with band-like infiltrate of lymphocytes in the papillary and intermediate dermis, well-differentiated infiltrating keratinizing SCC with individual cell dyskeratosis, and horn pearl formation; no evidence of vascular or perineural invasion (H&E stain 4.5×).

In terms of histological features, SCC arising in the context of LP retains the two primary characteristics of LP: basal epidermal keratinocyte damage and a lichenoid-interface lymphocytic reaction. However, SCC developing on this inflammatory lichenoid infiltrate shows also atypical keratinocytes with varying degrees of differentiation, ranging from well-differentiated areas with minimal pleomorphism, prominent keratinization (including parakeratosis), individual cell dyskeratosis, and horn pearl formation (Figure 4), to regions with pleomorphic nuclei, marked atypia, mitosis, and minimal keratinization. Additionally, the lichenoid inflammatory infiltrate can vary from focal areas to a dense, band-like distribution [43].

#### 3.2.3. Lichen Simplex Chronicus (LSC)

Lichen simplex chronicus (LSC) is a mucocutaneous disorder characterized by the thickening and lichenification of the skin or mucosa due to intense excoriation driven by excessive pruritus [44]. It involves approximately 12% of the general population, with the highest prevalence in adult patients between the ages of 30 and 50 years [45]. The most common sites affected by LSC, in decreasing order of incidence, include the scalp (particularly its posterior aspect), followed by the nape, ankles, vulva, scrotum, and extensor surfaces of the extremities [44,46].

LSC is classified into two forms: *primary* LSC, which develops de novo on otherwise normal-appearing skin or mucosa, often triggered by psychological or environmental factors; and *secondary* LSC, arising on pre-existing dermatological conditions, such as LP [46]. 

Given the pruritic nature of LP, LSC can often present as a long-term condition characterized by inflamed, leathery skin, although the degree of lichenification does not always correlate with the severity of itching [38]. LSC typically presents with erythematous, scaling, lichenified papules and plaques, sometimes excoriated. In long-standing cases, focal areas of hyperpigmentation and/or hypopigmentation may appear, along with prurigo nodularis (PN)-like lesions ranging from a few millimeters to 3 cm in size. These lesions are often bilaterally distributed on the trunk and extremities, tending to be larger and more difficult to treat in Black patients [46]. *Desquamation* is the most prominent clinical feature in nearly all cases.

**Figure 5:**
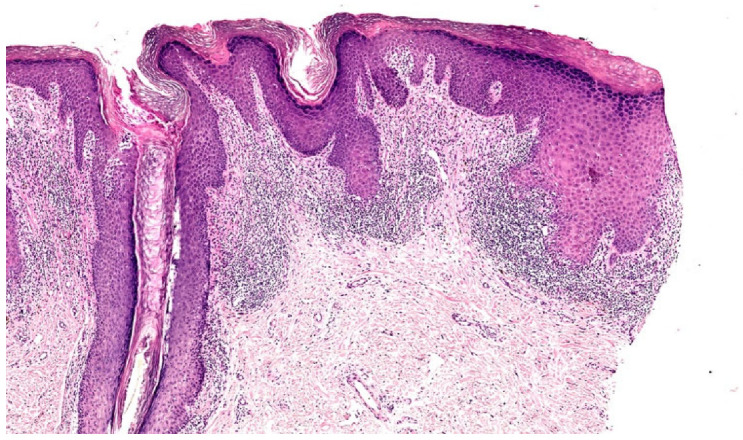
LSC superimposed on LP histopathology. Hyperkeratosis, focal desquamation, *not wedge-shaped* hypergranulosis, dense band-like lymphocytic infiltrate, vacuolar degeneration, and dyskeratosis at the DEJ and around the hair follicles (H&E stain 7×).

Regarding pathology, certain histological features of LSC are consistent: the *absence of wedge-shaped* hypergranulosis, greater regularity and uniformity in the length of the thickened dermal papillae, and less marked epidermal hyperplasia compared with hypertrophic lichen planus (HLP) [1,2,45] (Figure 5). In cases of PN superimposed on LP, hypergranulosis is observed, along with papillary dermal fibrosis and vertically oriented collagen fibers [47]. Additionally, a dissociation in nerve fiber density, with a reduction in intraepidermal fibers and an increase in dermal fibers, is characteristic [47].

#### 3.2.4. Pseudoepitheliomatous Hyperplasia (PEH)

Pseudocarcinomatous epidermal hyperplasia (PEH), also known as invasive acanthosis or carcinomatoid/verrucous epidermal hyperplasia, is a benign condition primarily characterized by epidermal and adnexal epithelial hyperplasia [48]. It can be diagnosed in isolation or associated with chronic inflammatory dermatoses and infections, such as Mycobacterium ulcerans, blastomycosis, and herpes simplex, particularly in immunocompromised individuals [49,50,51,52]. Oral lesions related to human papillomavirus (HPV) infection, particularly HPV subtypes 13 and 32, are referred to as Heck’s disease or focal epithelial hyperplasia [53]. Other HPV types, including HPV-6, -11, -16, and -18, have also been identified, with an increased prevalence in Native American populations [54]. 

Several primary cutaneous neoplasms, including lymphomas and carcinomas, may arise from PEH, often presenting as palpable, rapidly growing nodules [54,55]. Among inflammatory dermatoses, PEH has been associated with both mucosal LP and cutaneous hypertrophic lichen planus (HLP). 

Clinically, PEH typically presents as *well-defined*, *skin-colored*, tan, or pink plaques or nodules with varying degrees of scaling or crusting [55,56,57,58]. The prognosis of PEH is variable, as lesions may either regress spontaneously or increase in size, suggesting a potential malignant transformation, as seen in cases of transformed vulvar LP [59].

**Figure 6:**
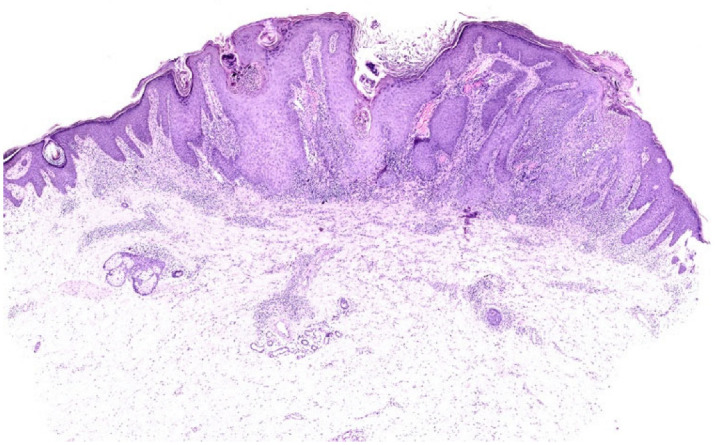
PEH associated with LP histopathology. Compact, basket-woven orthokeratosis, foci of hypergranulosis, severe epidermal hyperplasia with elongated thick downward projections of the epidermis, edema, and dilated blood vessels, and dense and band-like infiltrate of lymphocytes (H&E stain 6×).

Histologically, PEH is characterized by elongated, thick downward projections of the epidermis with jagged borders and a sharply pointed base. These projections represent the expansion of the epithelium and/or follicular infundibulum (pseudocarcinomatous hyperplasia), often extending to the upper or mid-reticular dermis [60] (Figure 6). Hypergranulosis and orthokeratosis or parakeratosis are commonly present. In some cases, concentric layers of abnormally shaped keratinocytes with central keratinization (keratin pearls) may be observed [60]. However, in contrast to squamous cell carcinoma (SCC), no evidence of cellular infiltration or necrosis, and moderate/severe keratinocyte atypia are reported. Mitotic figures are either rare or absent [55].

#### 3.2.5. Viral Warts

Human papillomavirus (HPV) is one of the most widespread viruses globally and the most common sexually transmitted viral infection [61]. It is frequently detected in various dermatologic conditions due to its ability to infect keratinocytes, leading to abnormal growths on the skin and mucous membranes. These include seborrheic keratoses, non-melanoma skin cancers (NMSCs), psoriatic plaques, and lichen planus (LP) [62]. 

Several studies have examined the potential role of HPV in oral LP (oLP), particularly in cases involving erosive oral lesions. HPV types 16 and 18 have been strongly associated with oLP, as the erosive nature of the lesions may facilitate the translocation of viral particles through the mucous membrane [63]. Although HPV DNA has been detected in some oLP cases, there is no conclusive evidence to suggest that HPV is directly involved in the etiopathogenesis of oLP [64]. Therefore, oLP is typically considered to be an autoimmune or hypersensitivity reaction rather than being caused by HPV.

HPV superimposing oLP should be suspected when oral lesions, typically presenting as whitish reticular, erosive, atrophic, papular, or plaque-like, progressively enlarge or develop small, finger-like projections resulting in *exophytic growths* [62]. 

**Figure 7:**
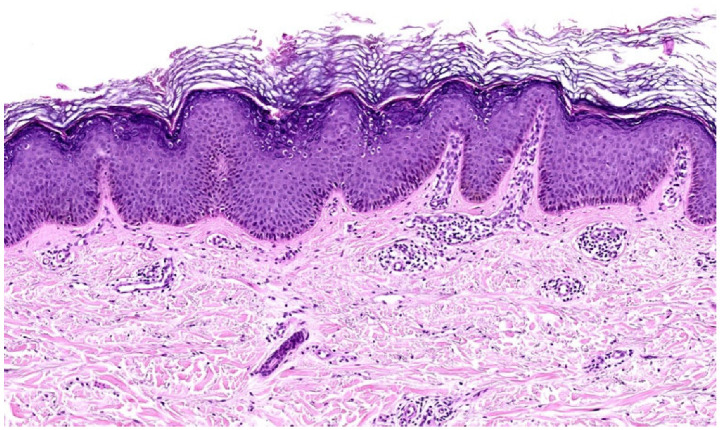
HPV infection without significant dermal lymphocytic band-like infiltrate histopathology. Orthokeratosis, focal hypergranulosis and parakeratosis, finger-like epidermal hyperplasia with keratinocytes containing rounded nuclei increased in size, and dilated blood vessels in the papillary dermis (H&E 15×).

The histopathology of HPV superinfection on LP, particularly in oLP, is distinctive and shows more pronounced changes than in cutaneous LP (cLP) [65]. This includes long, thin, finger-like projections of central connective tissue lined by stratified squamous epithelium. The presence of parakeratosis and koilocytes—large vacuolated cells with eccentric, hyperchromatic nuclei, and perinuclear cytoplasmic halos—is the most defining morphological feature of the cytopathic effects of HPV superinfection [65,66] (Figure 7).

While the correlation between cLP and HPV has been minimally explored, localized HPV superinfection on lichenoid plaques is theoretically possible, particularly in immunosuppressed patients [67].

#### 3.2.6. Lupus Erythematosus/Lichen Planus (LE/LP) Overlap Syndrome

LE/LP overlap syndrome is a rare condition that merges the clinical, histological, and immunopathological features of both disorders [68]. Over 60 cases of this overlap syndrome have been reported, primarily in individuals aged 17 to 71, with a slight female predominance [68,69,70,71,72,73,74,75,76,77,78]. A pediatric case has also been documented [79]. The syndrome is commonly characterized by *painful*, *centrally atrophic*, scaly, or verrucous plaques that can range in color from bluish red to pink or hypopigmented [72,73,75,80,81]. Additionally, papules may coalesce to form annular or polycyclic plaques [70]. Early-stage lesions often lack symptoms and the atrophic component, presenting as verrucous or papulonodular with more pronounced keratinization changes and epidermal hyperproliferation [70]. These lesions typically have a symmetrical distribution, often affecting the hands and arms. Less frequently, other areas such as the face, trunk, nails, mucous membranes, and scalp may be involved, with the scalp affected by *scarring alopecia* [73,82]. Palmoplantar involvement is also reported as a symptomatic manifestation [83]. The exact etiology remains unclear, but autoimmune, viral, and genetic factors are considered potential contributors [73]. Drugs such as isoniazid, procainamide, and acebutolol have also been identified as possible triggers [74]. 

**Figure 8:**
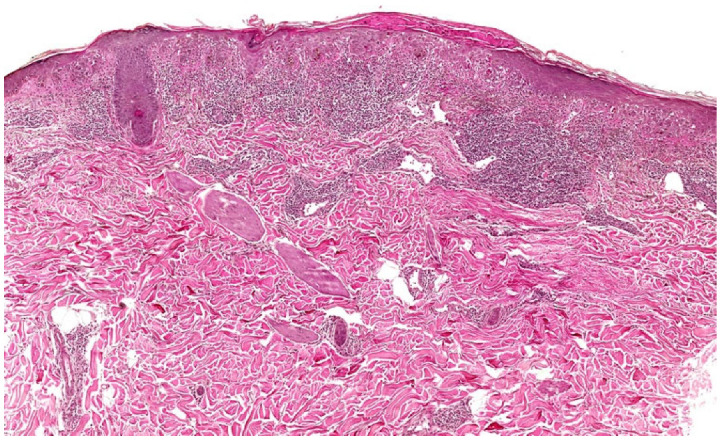
LE/LP overlap syndrome histopathology. Focal hyperplasia, parakeratosis, laminated orthokeratosis, thick basal cell layer, vacuolar alteration, and severe dermal perifollicular/perivascular and band-like inflammatory cell infiltrate at the DEJ (H&E 7.5×).

Histopathologically, LE/LP overlap syndrome is marked by severe dermal perifollicular and perivascular inflammatory cell infiltrates, with a band-like distribution at the dermal-epidermal junction. The presence of multiple lesion types within the same patient indicates the overlap nature of the syndrome (Figure 8). The histopathological features of LE include thickening of the basement membrane, hydropic degeneration of the basal layer without cleft formation, and chronic inflammation surrounding follicles and vessels [73]. In contrast, LP lesions typically show hyperkeratosis, hypergranulosis, irregular saw-tooth acanthosis, and pigment incontinence [8].

Currently, clear diagnostic criteria for LE/LP overlap syndrome have not been established. However, one proposed set distinguishes between “possible” and “classic” LE/LP overlap syndrome. The classic form is characterized by mixed clinical features of cutaneous lupus erythematosus (CLE) and LP, histological features consistent with LP (with or without accompanying features of CLE), and positive serologic markers for CLE [84].

## 4. Discussion

In everyday dermatological practice, the accurate diagnosis of skin and mucosal conditions often relies heavily on clinical evaluation. However, the prevalence of similar presentations among various dermatologic conditions—such as the hypertrophic clinical skin lesions defined by a histologic lichenoid infiltrate (HCLI)—requires skin biopsies to establish a definitive diagnosis and appropriate treatment. Although distinguishing between hypertrophic lichen planus (HLP) and lichen simplex chronicus (LSC) developing on a lichenoid infiltrate might seem less critical for therapy, it becomes important for prognosis. HLP tends to have a longer and more persistent course that requires different lines of treatment compared to LSC. Emerging treatment approaches are exploring the use of Janus kinase (JAK) inhibitors for HLP, whereas LSC may respond more readily to standard therapeutic interventions [7,85,86]. The differential diagnosis of HLP is extensive, and it is crucial to correlate clinical presentations with microscopic findings to identify the correct condition [87]. Several other less common conditions encountered in clinical practice or typically limited to a specific cutaneous extent should be considered. These include hypertrophic actinic keratosis (HAK), keratoacanthoma (KA), and hypertrophic lupus erythematosus (HLE). In HAK and KA, an underlying dermal lymphocytic infiltrate can indicate irritation or inflammation; KA is also defined by specific histologic features depending on its stage of development, from early proliferative to regressing stages. On the other hand, HLE, which can also present with pseudoepitheliomatous hyperplasia and vacuolar interface changes, has a dense band-like lymphocytic infiltrate, along with additional histologic features—such as follicular plugging, basement membrane thickening, and the presence of plasma cells—that help distinguish it from HLP [88,89,90]. In more difficult diagnostic cases of LE, the skin may exhibit an increased presence of CD123+ plasmacytoid dendritic cells, which serve as a valuable marker for identifying cellular clusters and supporting diagnosis [91].

The presence of pseudoepitheliomatous hyperplasia (PEH) in histological examination further complicates the diagnostic process, as it may resemble invasive carcinoma. This is particularly problematic when superficial biopsies fail to capture adequate dermal tissue, or when clinical presentation lacks distinctive features. PEH typically shows epithelial cell cords extending into the dermis [55], and while it may appear similar to invasive carcinoma, key distinguishing features include expanded follicular infundibula, broad rete ridges, and the lack of keratin pearls. These findings are often associated with underlying factors like infections, inflammation, or trauma, which are less commonly reported in squamous cell carcinoma (SCC) [92]. On the other hand, SCC displays nuclear atypia, necrotic keratinocytes, and mitotic figures. Immunohistochemical markers, such as increased p53-positive nuclear staining and elevated expression of matrix metalloproteinase-1 (MMP-1), can improve diagnostic accuracy and help differentiate SCC from PEH [92].

In cases where SCC resembles HLP, a study by Astudillo et al. identified several histopathological features that can aid in distinguishing between the two. For HLP, *wedge-shaped hypergranulosis* (*p* = 0.0033) and *irregular psoriasiform* hyperplasia (*p* = 0.004) were notable, while SCC presented with parakeratosis (*p* = 0.001), solar elastosis (*p* = 0.001), and perforating elastic fibers (*p* = 0.0001) as significant features [20]. Nevertheless, these characteristics are not definitive on their own, underscoring the importance of clinicopathological correlation in patients with atypical squamous proliferations, particularly those of the lower extremities.

SCC and oral lichen planus (oLP) can also present diagnostic and clinical challenges. Indeed, SCC that develops in the context of oLP often resembles conventional oral SCC, especially for localized anatomical sites. However, the lack of precursor lesions, such as leukoplakia or erythroplakia (commonly linked with conventional SCC), is a key distinguishing feature. Additionally, demographic data play an important role: patients experiencing malignant transformation from oLP are typically between the ages of 50 and 75, predominantly female, and often present with lesions on the buccal mucosa, lateral tongue, or gingiva. This contrasts with conventional SCC, which is more prevalent in males with a history of tobacco or alcohol use and typically appears on the tongue, followed by the gingiva and floor of the mouth [33]. This underscores the importance of taking the patient’s clinical history and presentation into account when diagnosing SCC in the context of oLP. Additionally, long-standing hypertrophic lichen planus (HLP) lesions should be closely monitored for signs of malignant transformation, such as easy bleeding or pain. These signs and/or symptoms may suggest the potential development of cutaneous SCC and warrant further investigation or intervention.

## 5. Conclusions

The hypertrophic skin lesions arising from a histological lichenoid infiltrate (HCLI) present a significant diagnostic challenge, even for experienced dermatologists and dermatopathologists. The absence of universally accepted and clearly defined pathological criteria for these conditions further complicates diagnosis. Nevertheless, key histological features must be carefully evaluated for accurate differentiation. (a) For LSC, the presence of regular, thickened papillary ridge morphology with non-wedge-shaped hypergranulosis. (b) For HLP, wedge-shaped hypergranulosis, irregular thickened papillary ridges, and well-differentiated squamous epithelium confined to the superficial dermis. (c) For PEH, irregular epidermal hyperplasia and mild dyskeratosis. (d) For SCC, atypical keratinocytes, and mitotic figures. (e) For HPV-induced lesions on a lichenoid infiltrate, parakeratosis and koilocytes. (f) For LE/LP overlap syndrome, basement membrane thickening, inflammatory cell infiltrates involving the dermal follicles and vessels, shaggy fibrinogen deposition and granular layering of immunoglobulin and complement along the dermoepidemal junction.

The careful selection of the biopsy site and technique is essential to avoid superficial sampling, thereby improving diagnostic accuracy and ensuring the correct identification of the histological features of each hypertrophic condition.

## Figures and Tables

**Table 1:** Clinical, histopathological, and prognostic features of hypertrophic clinical lesions associated with band-like lichenoid infiltrate (HLCI).

HCLI	Clinic *	Histopathology					Prognosis
		Type of Hypergranulosis	Epidermal Hyperplasia	Papillary Ridge Morphology	Band-Like Infiltrate of the Lymphocytes	Other Specific	
HLP	Thickened, verrucous, purple-red, plaque/nodule	Wedge-shaped	Pseudoepitheliomatous hyperplasia, endophytic well-differentiated squamous epithelium extending not beyond the superficial dermis	Irregular	Present	Basal cell vacuolar alteration; thickening of collagen fibres in the papillary dermis	Longer mean duration and often unremitting compared with LP
HLS	Whitish plaque variably combining thickness and atrophic appearance	Wedge-shaped	Hyperkeratosis, atrophic and irregular thickening of the epidermis	Irregular	Present (below the zone of hyalinization)	A broad, pale, eosinophilic band of collagen (hyalinized tissue) in the upper dermis, vascular changes, basal vacuolization, pigmentary incontinence HLS without sclerosis: Lymphocytes and necrotic keratinocytes scattered throughout the epidermis, psoriasiform hyperplasia, Civatte bodies, alignment of lymphocytes at the basal layer	Poor response to therapy
Hypertrophic lichenoid dermatitis	Thickened, erythematous, scaly, plaque/nodule	Wedge-shaped	Hyperkeratosis, orthokeratosis	Regular/irregular	Present	Prominent spongiosis, eosinophilic infiltrate, generalised distribution	Discontinuation of immune checkpoint inhibitors is usually not required
SCC	Skin Erythematous-violaceous plaque with a tendency to ulcerate and/or depigment Mucosa Whitish surface with elevated indurated borders; exophytic or endophytic growth patterns with subsequent ulcer formation	Wedge/not wedge-shaped	Dyskeratosis, orthokeratosis	Irregular	Present	Atypical keratinocytes with a variable degree of differentiation: from horn pearl formations to a high number of mitoses and few areas of keratinisation	Depending on staging: locally advanced and rarely metastatic
KA	Dome-shaped tumour capped with keratin. Giant (>5cm) lesions have a predilection for the nose and the dorsum of the hands	Wedge/not wedge-shaped	Dyskeratosis and orthokeratosis (evident in the keratin-filled central crater)	Irregular	Present	Expansion of squamous epithelium forming irregular epithelial proliferations with central keratinization. (Early proliferative stage) Crateriform architecture, epidermal lipping on both sides of the keratin core (Mature stage) Lesions become thinner/flattened, with fewer squamous lobules and horn pearl, dermal scar-like fibrosis (Regressing stage)	Potential involution in several months
LSC	Scaling lichenified with overlying excoriation, areas of hyper/hypopigmentation	Not wedge-shaped	Severe	Regular/irregular	Present	Increased fibrocyte number; thickened papillary dermis	Good response to therapy
PEH	Well-demarcated plaque/nodule	Wedge/not wedge-shaped	Elongated projections of the epidermis starting from the follicular infundibulum, ortho/parakeratosis, *keratin pearls*	Regular, elongated	Present	Few mitotic figures without atypia/with mild atypia	Variable: increasing in size or spontaneous regression
Viral warts	Papule defined by a verrucous surface	Focal hypergranulosis	Hyperkeratosis, orthokeratosis	Elongated	Present	*Koilocytes* and dilated blood vessels in the papillary dermis	Worse in immunocompromised patients
LE/LP overlap	Painful, bluish-red or hypopigmented plaque acquiring a verrucous (wart-like) appearance	Wedge/not wadge-shaped	Focal hyperplasia, parakeratosis, *and laminated* orthokeratosis	Irregular, thin	Presently, with significant perifollicular and perivascular inflammatory cell infiltrate	Vacuolar alteration at the DEJ, thick basal cell layer PAS positive	Variable

* Unless otherwise specified, the clinical presentation of lichen planus on the skin is typically the same as that observed on mucosal sites. DEJ (Dermoepidemal junction), HLP (Hypertrophic Lichen Planus), HLS (Hypertrophic Lichen Sclerosus), KA (Keratoacanthoma), LE/LP (Lupus Erythematous/Lichen Planus) overlap, LSC (Lichen Simplex Chronicus), PAS (Periodic Acid-Schiff), PEH (Pseudoepiteliomatous hyperplasia), SCC (Squamous Cell Carcinoma).

## Data Availability

The data presented in this study are both available in the literature and from the corresponding author upon reasonable request.
